# Tracheal Tube Misplacement after Emergency Intubation in Pediatric Trauma Patients: A Retrospective, Exploratory Study

**DOI:** 10.3390/children9020289

**Published:** 2022-02-18

**Authors:** Franziska Rost, Bernd Donaubauer, Holger Kirsten, Thomas Schwarz, Peter Zimmermann, Manuela Siekmeyer, Daniel Gräfe, Sebastian Ebel, Christian Kleber, Martin Lacher, Manuel Florian Struck

**Affiliations:** 1 Department of Anesthesiology and Intensive Care Medicine, University Hospital Leipzig, 04103 Leipzig, Germany; franziska.rost@medizin.uni-leipzig.de (F.R.); bernd.donaubauer@medizin.uni-leipzig.de (B.D.); thomas.schwarz1@med.uni-goettingen.de (T.S.); 2 Institute for Medical Informatics Statistics and Epidemiology, Medical Faculty Leipzig, 04107 Leipzig, Germany; holger.kirsten@imise.uni-leipzig.de; 3 Department of Anesthesiology, University Medical Center Göttingen, 37075 Göttingen, Germany; 4 Department of Pediatric Surgery, University Hospital Leipzig, 04103 Leipzig, Germany; peter.zimmermann@medizin.uni-leipzig.de (P.Z.); martin.lacher@medizin.uni-leipzig.de (M.L.); 5 Pediatric Intensive Care Unit, Department of Pediatrics, University Hospital Leipzig, 04103 Leipzig, Germany; manuela.siekmeyer@medizin.uni-leipzig.de; 6 Institute of Pediatric Radiology, University Hospital Leipzig, 04103 Leipzig, Germany; daniel.graefe@medizin.uni-leipzig.de; 7 Department of Diagnostic and Interventional Radiology, University Hospital Leipzig, 04103 Leipzig, Germany; sebastian.ebel@medizin.uni-leipzig.de; 8 Department of Orthopedics, Traumatology and Plastic Surgery, University Hospital Leipzig, 04103 Leipzig, Germany; christian.kleber@medizin.uni-leipzig.de

**Keywords:** pediatric trauma, tracheal intubation, airway management, helicopter emergency medical service

## Abstract

Inadvertent tracheal tube misplacement and particularly endobronchial intubation are well-known complications of emergency endotracheal intubation (ETI) in pediatric trauma patients, which require repositioning of the tube to avoid impairment of gas exchange. The main aim of study was to identify the frequency of tube misplacement and associated factors of pediatric trauma patients who received ETI either by prehospital physician-staffed emergency medical service (EMS), or at emergency department (ED) admission to a single level-1 trauma center. Sixty-five patients (median age 14 years and median injury severity score 29) were included. Of these, 30 underwent helicopter EMS ETI, 29 ground EMS ETI, and 6 ED ETI. Seventeen cases (26%) of tracheal tube misplacement were recognized. After multivariable analysis, tracheal tube misplacement was independently negatively associated with body weight (OR 0.86; 95% CI, 0.76–0.99; *p* = 0.032) and helicopter EMS ETI (OR 0.20; 95% CI, 0.04–0.97; *p* = 0.036). Two of nineteen patients received tube thoracostomy due to endobronchial intubation. Mortality and length of stay were comparable in patients with misplaced tubes and correctly placed tubes. The results suggest that particularly small children require attention to avoid tracheal tube misplacement, which emphasizes the need for special training. Helicopter EMS physicians’ expertise might be beneficial in prehospital pediatric trauma patients requiring advanced airway management.

## 1. Introduction

Advanced airway management in pediatric patients is a challenge for emergency medical service (EMS) providers because of the considerable physiological and anatomical differences and a much lower frequency compared to adults [1,2,3]. In the United States, the estimated pediatric out-of-hospital tracheal intubation rate is 6.7 per 1000 cases [1]. According to a recent meta-analysis, the risk of intubation failure is 3.54-fold higher in pediatric patients than in adults [4]. Success rates vary considerably depending on the EMS system, whereas physician-staffed providers, particularly physician-staffed helicopter EMS, may have higher expertise than paramedic-based systems [5,6,7,8,9]. 

Unrecognized esophageal intubation accounts for the most fatal mechanical complication, ultimately leading to hypoxia and death. Tracheal intubation into one mainstem bronchus may cause impairment of gas exchange and lung atelectasis [10]. Furthermore, unrecognized endobronchial intubation may also lead to avoidable chest tube placement at the unventilated chest side due to absent breath sounds mimicking pneumothorax [11,12]. Although there is previous evidence that children are prone to endobronchial intubation, studies identifying associated factors are scarce [12,13,14,15,16,17,18,19,20,21]. 

Thus, the aim of this study was, first, to analyze the frequency of tracheal tube misplacement in a study cohort of pediatric trauma patients that had intubation performed by either prehospital EMS or in the emergency department of a tertiary care hospital, and second, to identify associated factors and clinical consequences of this complication. 

## 2. Materials and Methods

### 2.1. Study Design

Data from pediatric trauma patients who underwent prehospital or emergency department intubation were retrospectively analyzed regarding the need for tracheal tube replacement due to misplacement and particularly endobronchial intubation (the raw data can be found in Table S1). The analysis of associated factors with tube misplacement included demographic data, management characteristics, and outcomes of patients, comparing cases with correctly placed tubes and cases with misplaced tubes. Furthermore, we investigated all patients who underwent tube thoracostomy, particularly those with tracheal tube misplacement. This study was approved by the Ethics Committee of the Medical Faculty of Leipzig, Germany (No. 441/15-ek), which waived the need for informed consent. The study was retrospectively registered at the German Clinical Trials Register under DRKS-ID: DRKS00028045.

### 2.2. Patient Enrollment

The local trauma registry of the University Hospital Leipzig was retrospectively reviewed for pediatric trauma patients admitted from January 2008 to December 2019. The inclusion criteria were age < 18 years, admission directly from the scene of the accident, and emergency tracheal intubation in the prehospital setting at the scene of the accident or during management in the emergency department. Patients with supraglottic airway devices were excluded. Tracheal tube malposition requiring replacement was defined as unrecognized esophageal intubation, endobronchial intubation (detected on CT scans, plane chest radiography, and by auscultation), and tube dislocations or critical tube-to-carina distances with tube positions less than two centimeters above the carina (identified on either CT scans or plane chest radiography). Data were obtained from medical records, the radiological information system, and the picture archiving and communication system (MEDOS RIS version 9.3.3008, Nexus MagicWeb Version VA60C_0115, Visage Imaging, PACS: syngo.plaza, Siemens Healthcare, Erlangen, Germany).

### 2.3. General Management

Prehospital care of pediatric major trauma patients was performed by emergency response physicians until hospital admission. In the City of Leipzig and the greater Leipzig area, there were 19 physician-staffed ground EMS bases and 4 helicopter EMS bases. One ground EMS base was stationed at the study hospital and was exclusively run by their physicians. These physicians mainly worked in departments of anesthesiology, trauma surgery, or emergency department and attended 2–4 shifts per month at the EMS base. Two helicopter EMS of the Leipzig region were staffed with physicians from the study center and two other tertiary care hospitals. Airway management equipment of all prehospital EMS providers contained direct laryngoscopy devices (Macintosh blades), supraglottic airway devices (laryngeal tubes or laryngeal masks), and surgical airway devices (percutaneous devices using the Seldinger technique, or open cricothyroidotomy). Video laryngoscopy devices (including Macintosh-style blades and hyper-angulated blades) were available on two physician-staffed EMS helicopters in the greater Leipzig area, whereas most ground-based EMS providers had also introduced them in their physician-staffed rapid response units. Airway management equipment of the emergency department was comparable to EMS devices and additionally included fiberoptic bronchoscopes for awake fiberoptic intubation.

Emergency department and trauma room management were organized according to the recommendations of the German Society of Pediatric Surgery (DGKCH) and the German Society of Trauma Surgery (DGU) [22,23]. The trauma room activation was performed by the EMS teams at the scene or by a dispatcher of the Leipzig Fire Control Center. After standardized clinical assessment and under consideration of individual condition, emergency sonography and an initial whole-body CT were performed, whereas critically unstable children were transferred directly to the operating room or underwent only head CT. In children who underwent chest CT or plane chest radiography (CXR), tracheal tube positions were classified according to tube-to-carina distances (TCDs), where available. TCD was measured using the RIS/PACS.

### 2.4. Statistical Analysis

The data are reported as the mean (standard deviation (SD)) for normally distributed data, as the median (interquartile range (IQR)) for non-normally distributed data, and as numbers (percentages). Patient characteristics were compared by applying Fisher’s exact test and the Mann–Whitney U test or Student’s *t*-test for non-normally and normally distributed variables, respectively. Odds ratios (ORs) and 95% confidence intervals (CIs) were calculated in the framework of logistic regression analysis, comparing correctly placed tubes and tubes requiring replacement. The investigated associations for tubes requiring replacement were age, sex, height, weight, body mass index (BMI), injury severity score (ISS), admission systolic blood pressure (SBP) values, Glasgow coma scale (GCS) scores, SpO_2_ values, American Society of Anesthesiologists (ASA) classification, chest CT or plain chest radiography (CXR) data, and performances of emergency surgery and cardiopulmonary resuscitation (CPR). Age groups were infants (<1 year), pre-school children (1–5 years), schoolchildren (6–11 years), and adolescents (12–17 years). The investigated outcome factors were length of stay in the pediatric intensive care unit (PICU), ventilator days, 24 h mortality, and 30-day mortality. Data on procedure details (intubation situs and complications) and qualification levels of providers were primarily collected. In case the number of missing data on these variables was high, no further analysis was performed. The alpha level of significance was set at 0.05 without correction for multiple testing as we followed an exploratory approach. All tests were two-tailed. Multivariable analysis was performed on parameters found to be statistically significant in univariable analysis (*p* ≤ 0.05) to assess independent associations applying stepwise logistic regression based on Akaike’s information criterion, as implemented in the stepAIC function of the R addon package MASS 7.3.54. All analyses were performed in the framework of R 4.1.1.

## 3. Results

### 3.1. Baseline Characteristics

**Figure 1:**
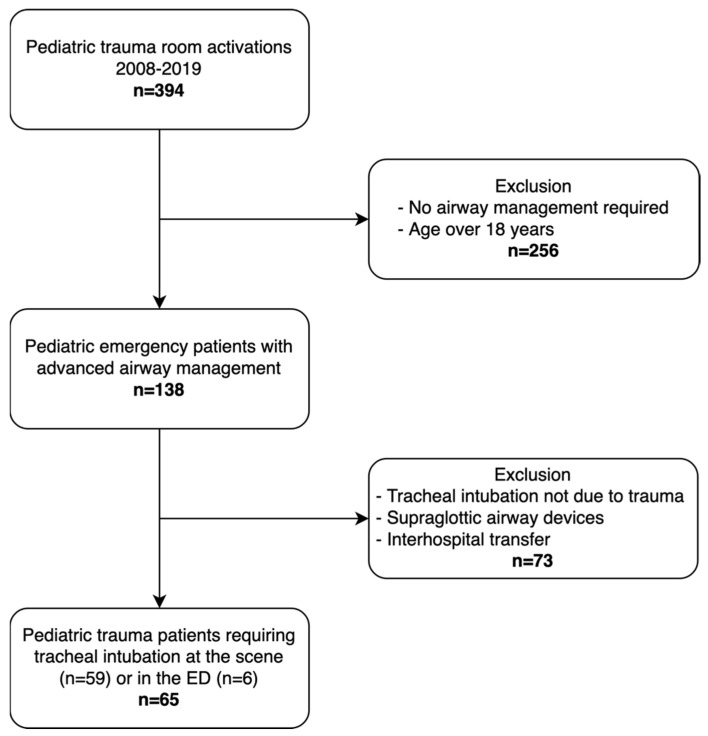
Study flowchart.

Sixty-five children met the inclusion criteria and were analyzed (Figure 1). The baseline characteristics are displayed in Table 1. Median patient age was 14 years (IQR 8.5). Patients’ ages were distributed as follows: infants under one year: none (0.0%), preschool children (1 to 5 years): 12 (18.5%), schoolchildren (6–11 years): 14 (21.5%), and adolescents (12–18 years): 39 (60.0%). The blunt trauma mechanism was exclusively recorded (61.5% road traffic accidents, 23.1% falls from height, and 15.4% blunt trauma of other causes). The median ISS was 29 (IQR 21). Tracheal intubation was attempted in the prehospital setting in 59 patients (90.8%; 30 by helicopter EMS and 29 by ground EMS) and in the emergency department in 6 patients (9.2%). After intubation, all patients received capnography monitoring to confirm the tracheal tube position.

### 3.2. Tracheal Intubation

Seventeen cases (26.2%) of tracheal tube misplacement were recognized within the first hour of acute trauma resuscitation, of which eight were endobronchial intubations (five detected on CT scans and three by auscultation). Nine cases were tube dislocations or critical tube-to-carina distances with tube positions less than 2 cm above the carina, including one case of esophageal intubation that was immediately recognized and corrected in the prehospital setting and presented with a tube position of 0.5 cm above the carina on CT. Cases of unrecognized esophageal intubation were not observed.

**Figure 2:**
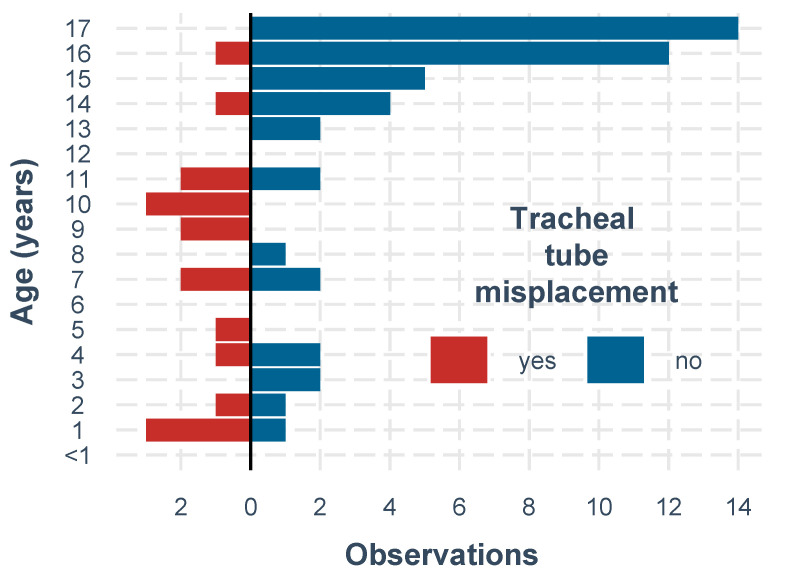
Tracheal tube misplacement and age.

ISS, sex, and vital parameters were similarly distributed in both study groups, whereas age, height, weight, BMI, and helicopter EMS ETI provided significant differences of tracheal tube misplacements (Figure 2). Regarding the method of intubation, patients requiring CPR at the scene underwent crash ETI without the use of drugs, whereas all other patients received rapid sequence induction ETI. Patients with and without CPR presented with comparable ETI misplacement rates.

### 3.3. Risk Factors Associated with Tracheal Tube Misplacement

**Figure 3:**
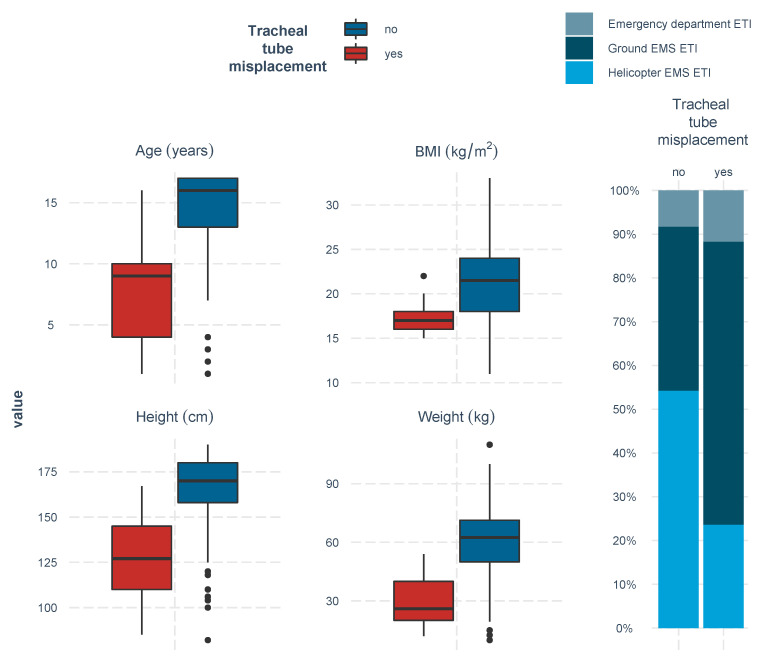
Univariable associations with tracheal tube misplacement.

Univariable risk factors (negatively) associated with tracheal tube misplacement were age, body height, body weight, body mass index, and helicopter EMS ETI (Table 2, Figure 3). After adjustment using multivariable stepwise logistic regression analysis removing age and body mass index (as being part of the variables of height and weight), tracheal tube misplacement remained independently negatively associated with body weight (OR 0.86; 95% CI, 0.76–0.99; *p* = 0.032) and helicopter EMS ETI (OR 0.20; 95% CI, 0.04–0.97; *p* = 0.036) (Table 3). 

### 3.4. Characteristics of Helicopter EMS and Ground EMS

A detailed analysis of prehospital EMS characteristics confirmed significantly higher misplacement rates (*p* = 0.039) and further revealed significantly lower Glasgow coma scale scores (*p* = 0.043), higher injury severity scores (*p* = 0.034), and most significantly, lower systolic blood pressure values (*p* = 0.003) in patients intubated by ground EMS rather than by helicopter EMS. Although the rate of cardiopulmonary resuscitation prior to ED admission and 24 h mortality did not reach significant levels in the ground EMS ETI group, a significantly higher 30-day mortality rate was observed (*p* = 0.047) (Table 4). 

### 3.5. Clinical Consequences of Tracheal Tube Misplacement 

Nineteen patients received tube thoracostomy, of which two had confirmed endobronchial intubation and no radiological confirmation of thoracic injury. In these two patients, tube thoracostomy was performed due to absent breathing sounds, which were erroneously considered as pneumothorax. Mortality rates after 24 h and 30 days, length of stay in the PICU, ventilator days, emergency surgery, and CPR frequencies were comparable in patients with and without ETI misplacement.

## 4. Discussion

The observed frequency of tracheal tube misplacement of 26.0% and particularly 12.3% endobronchial intubation in our study is in line with the results of previous pediatric studies. In the last two decades, various frequencies of endobronchial tube misplacements, ranging from 0% [8], over 2.4% [3], 3.6% [14], 6.2% [19], 6.4% [18], 8% [17] 12.8% [16], 13.2% [21], to 21.0%, have been reported [12]. Furthermore, some studies assessing pediatric tube positions found that the tube tips were generally placed too deep in even larger proportions (24.5% [16], 38.3% [20], 38.9% [15], 50.0% [12], and 69% [24], respectively).

Univariable analysis of our data revealed the patient-dependent variables of young age, short body height, low body weight, and low body mass index as being associated with tracheal tube misplacement. After the adjustment for multivariable testing, a significant association between low body weight and tracheal tube misplacement remained evident. Low body weight as a risk factor for tracheal tube misplacement in pediatric trauma patients is a new finding that has to be confirmed in further studies. These studies should provide relevant sample sizes across all age categories to rule out whether our findings are center-specific or may be generalizable.

There are some anatomical factors that may contribute to tracheal tube misplacement. First, the quality of the tube’s fixation may contribute to its dislocation. Second, the documentation of the insertion depth itself, measured by the reading of the tube at the fixation at the incisors or the mouth’s angle, is crucial to assess the risk of tube malposition. In children in whom tracheal tube displacement was only recognized after arrival in the emergency department, it cannot be determined whether the tube was originally placed incorrectly or if it dislocated during transport [25]. 

Numerous studies investigated the impact of head movement after intubation on the TCD and on the tube tip-to-vocal cords distance, respectively. Neck flexion results in moving the tracheal tube towards the carina, while neck extension leads to a displacement in the opposite direction. The rotation of the head to the side can withdraw the tube tip from the trachea towards the tube fixation or away from the tube fixation, respectively [26,27,28,29]. Considering these findings, it seems reasonable that once correctly placed ETTs can easily displace, particularly in smaller children.

An improved tube insertion depth may be achieved by assessing various anatomical distances (e.g., the upper incisor to manubriosternal joint length) prior to intubation [30,31,32]. Formula approaches on bedside prediction of adequate tube insertion depth use body weight, body height, tube diameter (e.g., 3× tube size), and the age in years for calculations [33,34,35]. However, in an emergency situation, the actual height and weight of the patients are often not available and have to be roughly estimated. Thus, only capnography and the visual placement of the tube marking between the vocal cords may be reliably associated with a correct tracheal positioning of the tube tip [36]. The individual anatomy (e.g., a short neck) always needs to be considered for the prevention of deep tube misplacements.

Regarding the frequency of tube misplacement comparing helicopter EMS and ground EMS, we observed a beneficial effect of helicopter EMS that remained after adjustment with other significant patient-related variables. Although both EMS systems were physician-staffed, helicopter EMS usually provide more experienced doctors than ground EMS. For instance, at the two helicopter EMS bases mainly responsible for the catchment area of the study, physicians needed to have a board certification (anesthetists in most cases) and a professional experience of more than five years in ground EMS. However, patients intubated by ground EMS were significantly more severely injured than helicopter EMS patients. This may impair the comparability and interpretation of the results and should be noted. In ground EMS intubation, the CPR rate until ED admission was almost twice as high as in helicopter EMS intubation. Since intubation under CPR is usually performed as crash intubation, emergency circumstances may have also contributed to a higher tube misplacement rate in this group. We considered chest compression-associated movements under CPR as a possible contributing factor for tube misplacement, but it did not reach statistical significance.

In the literature, recent studies involving greater sample sizes of pediatric patients found high rates for overall and first-pass intubation success, and even survival benefits in helicopter EMS [5,6,7,13,14,37,38,39,40,41]. One study found no difference in mortality comparing helicopter EMS and ground EMS, although helicopter EMS patients had a higher injury severity [42]. Furthermore, there is a controversy in the literature regarding air transport vs. ground transport in children with minor vs. major injuries [41]. In summary, our results support previous studies in that, whenever available, helicopter EMS should be dispatched simultaneously with ground EMS to achieve the best possible intubation performances in pediatric trauma patients requiring advanced airway management [4,5,6].

Our results suggest that pediatric trauma patients experiencing tracheal tube misplacement are not associated with adverse outcomes compared with patients with correctly placed tubes. Mortality rates at 24 h and at 30 days were similar in all groups. This is in line with results from 26 of 616 intubated adult trauma patients of our center [11]. However, we observed two cases of possibly unnecessary tube thoracostomy due to unrecognized endobronchial intubation [12]. Thus, we recommend routine use of point-of-care ultrasound (POCUS) to exclude pneumothorax in prehospital and emergency department trauma management [43].

After emergency intubation of a severely injured child, we recommend the thorough fixation of the tube after auscultation and capnography, inspection of the chest for breathing symmetry, and POCUS assessment, if available. The insertion depth of the tube (in cm) should be written at the fixation material and documented in the charts. In case of massive facial or oral bleeding, pharyngeal tamponade may be applied. The head should be fixed (e.g., head blocks, cervical spine immobilization) and the tracheal tube and ventilation hose should be positioned without any tension to prevent dislocation due to pull-forces during EMS transport and handover maneuvers. During transport, frequent inspection of the tube insertion site and, if possible, repeated auscultation of the chest should be performed. If the respiratory and/or circulatory condition deteriorates, kinking of the ventilator hose or tracheal tube should be checked and correct tube connection should be confirmed [44]. Possible pneumothorax should be anticipated early and ultimately be decompressed.

### Limitations

We are aware of several limitations of the study, including the retrospective design. The relatively small sample size and the single center design lower the significance of our results. Not all of our patients underwent CT scans following tracheal intubation and patients who died at the scene or were declared dead on arrival were not included and might have presented with other characteristics. A detailed analysis of the airway management (e.g., intubation situs, number of attempts or complications, training level of intubating physician) was not possible due to inconsistent documentation or missing data. Another limitation is that we did not document tube insertion depth on admission to the emergency department. Furthermore, our data analysis followed an explanatory approach. Hence, our findings require replication in independent datasets. 

## 5. Conclusions

The results of this study cohort suggest that particularly small children require attention to avoid tracheal tube misplacement, which emphasizes the need for special training. Helicopter EMS physicians´ expertise might be beneficial in prehospital pediatric trauma patients requiring advanced airway management, which has to be confirmed in further prospective studies with higher sample sizes.

## Figures and Tables

**Table 1:** Baseline characteristics of the study cohort.

	Overall	Tracheal Tube Position Correct	Tracheal Tube Misplacement	*p*
*n* (%)	65 (100)	48 (73.8)	17 (26.2)	
Age (years)	14.0 (8.5)	16 (4)	9 (7.5)	<0.001
Male, *n* (%)	40 (62)	31 (65)	9 (53)	0.404
Height (cm)	165 (48)	170 (22)	127 (42)	<0.001
Weight (kg)	51.2 (25.1)	59.2 (23.5)	28.7 (13.1)	<0.001
BMI	20.4 (4.7)	21.5 (4.8)	17.7 (2)	<0.001
GCS	8 (8)	8 (10.5)	4 (7)	0.317
SBP (mmHg)	100 (85.5)	100 (78.5)	90 (110)	0.208
HR (min^−1^)	100 (37.5)	102.5 (44.5)	98 (77)	0.153
SpO_2_ (%)	95 (18)	93 (17.3)	97 (52)	0.116
ISS	29 (21)	26 (19.5)	29 (27)	0.373
RTA, *n* (%)	40 (61.5)	30 (62.5)	10 (58.8)	0.781
Fall from height, *n* (%)	15 (23.1)	11 (22.9)	4 (23.5)	1
Other cause *n* (%)	10 (15.4)	7 (14.6)	3 (17.6)	0.713
Helicopter EMS ETI, *n* (%)	30 (46.2)	26 (54.2)	4 (23.5)	0.046
Ground EMS ETI, *n* (%)	29 (44.6)	18 (37.5)	11 (64.7)	0.087
ED ETI, *n* (%)	6 (9.2)	4 (8.3)	2 (11.8)	0.648
CPR prior ED, *n* (%)	19 (29)	13 (27)	6 (35)	0.546
WBCT, *n* (%)	43 (66.2)	33 (68.8)	10 (58.8)	0.554
CCT only	10 (15.3)	6 (12.5)	4 (23.5)	0.434
No CT	12 (18.5)	8 (16.7)	4 (23.5)	0.713
Emergency surgery, *n* (%)	46 (70.8)	37 (66.7)	9 (52.9)	0.071
Ventilator (days)	1.0 (5)	1.0 (5)	1 (6)	0.596
PICU (days)	5.0 (11.5)	5.0 (11.5)	6 (12)	0.611
24 h mortality, *n* (%)	8 (12.3)	5 (10.4)	3 (17.6)	0.421
30-day mortality, *n* (%)	18 (27.7)	12 (25.0)	6 (35.3)	0.529

BMI, body mass index; GCS, Glasgow coma scale; SBP, systolic blood pressure; HR, heart rate; SpO_2_, peripheral oxygen saturation; ISS, injury severity score; RTA, road traffic accident; EMS, emergency medical service; ETI, endotracheal intubation; ED, emergency department; CPR, cardiopulmonary resuscitation; WBCT, whole-body computed tomography; CCT, cranial computed tomography; CT, computed tomography; PICU, pediatric intensive care unit. Squared brackets indicate interquartile ranges (IQR) preceded by medians, while round brackets of continuous traits indicate standard deviations, preceded by means.

**Table 2:** Significant univariable risk factors associated with tracheal tube misplacement.

Predictor	Univariable OR (95% CI)	*p*
Age	0.81 (0.71–0.90)	<0.001
Height	0.96 (0.94–0.98)	<0.001
Weight	0.94 (0.90–0.97)	<0.001
BMI	0.75 (0.61–0.89)	0.003
Helicopter EMS ETI	0.26 (0.06–0.85)	0.035

BMI, body mass index; EMS, emergency medical service; ETI, endotracheal intubation.

**Table 3:** Significant adjusted predictors identified in a stepwise logistic multivariable regression model of associated factors with tracheal tube misplacement.

Predictor	Multivariable OR (95% CI)	*p*
Weight	0.86 (0.76–0.99)	0.032
Helicopter EMS ETI	0.20 (0.04–0.97)	0.036

EMS, emergency medical service; ETI, endotracheal intubation.

**Table 4:** Comparison of helicopter EMS vs. ground EMS characteristics of the study cohort.

	Helicopter EMS ETI	Ground EMS ETI	*p*
*n* (%)	30 (56.2)	29 (44.6)	
Age (years)	14.5 (6)	16 (8)	0.681
Male, *n* (%)	19 (63.3)	19 (65.5)	1
Height (cm)	155.7 (27.2)	152.8 (31.3)	0.968
Weight (kg)	52.6 (21.8)	54.6 (27.7)	0.652
BMI	20 (5)	21 (8)	0.575
GCS	8 (11)	5 (7)	0.043
ISS	25 (18)	34 (20)	0.034
SBP (mmHg)	111 (52)	80 (110)	0.003
HR (min^−1^)	108.5 (35)	98 (59)	0.067
SpO_2_ (%)	94.5 (10)	94 (77)	0.435
ETI misplacement, *n* (%)	4 (13.3)	11 (37.9)	0.039
CPR prior ED, *n* (%)	6 (20)	11 (37.9)	0.158
24 h mortality, *n* (%)	2 (6.7)	5 (17.2)	0.254
30-day mortality, *n* (%)	5 (16.7)	12 (41.4)	0.047

EMS, emergency medical service; ETI, endotracheal intubation; BMI, body mass index; GCS, Glasgow coma scale; ISS, injury severity score; SBP, systolic blood pressure; HR, heart rate; SpO_2_, peripheral oxygen saturation; CPR, cardiopulmonary resuscitation; ED, emergency department; WBCT, whole-body computed tomography. Squared brackets indicate interquartile ranges (IQR) preceded by medians, while round brackets of continuous traits indicate standard deviations, preceded by means.

## Data Availability

Data supporting the reported results can be found in Table S1: Raw data file.

## Supplementary Materials

The following supporting information can be downloaded at: https://www.mdpi.com/article/10.3390/children9020289/s1, Table S1: Raw data file.
